# Idiopathic anterior herniation of the thoracic cord: a review

**DOI:** 10.1055/s-0045-1812297

**Published:** 2025-11-28

**Authors:** Ana Cristina Veiga Silva, Raívson Diogo Félix Fernandes, Emanuella Arruda do Rêgo Nóbrega, Joaquim Fechine de Alencar Neto, Otávio da Cunha Ferreira Neto, Rocymar Rebouças Oliveira Júnior, Lidemarks Irineu Andrade, Rita de Cassia F. Valença Mota, Antônio Rodrigues de Aguiar Neto, Geraldo de Sá Carneiro Filho, Deoclides Lima Bezerra Junior, Hildo Rocha Cirne de Azevedo Filho

**Affiliations:** 1 Hospital da Restauração, Departamento de Neurocirurgia, Recife PE, Brazil.; 2 Centro Universitário Unifacisa, Faculdade de Ciências Médicas, Campina Grande PB, Brazil.; 3 Universidade de São Paulo, Faculdade de Medicina de Ribeirão Preto, Hospital das Clínicas, Departamento de Cirurgia e Anatomia, Divisão de Neurocirurgia, Ribeirão Preto SP, Brazil.; 4 Universidade Federal de Pernambuco, Programa de Pós-Graduação em Neurociências, Recife PE, Brazil.

**Keywords:** Hernia, Idiopathic, Review, Spinal Cord, Spinal Cord Diseases, Thoracic Vertebrae

## Abstract

Idiopathic spinal cord herniation (ISCH) is a rare condition caused by a defect in the dura mater, resulting in ventral displacement of the spinal cord. Its etiology is not fully understood, but it mainly affects middle-aged women and manifests as progressive myelopathy. Surgical treatment is the best option to avoid neurological worsening. This report presents a case of spinal cord herniation in a 45-year-old man, complaining of numbness for 4 months, with paraparesis that progressed to gait disorder. This condition was diagnosed by magnetic resonance imaging and computed tomography. The patient underwent T2 to T3 laminectomy, hernia reduction, and duroplasty, with successful resolution of the condition.

## INTRODUCTION

Idiopathic spinal cord hernia (ISCH) is a condition characterized by ventral displacement of spinal cord fibers, through a failure in the dural structure, whether congenital or acquired. 1 Its etiology is the subject of extensive debate but the trigger mechanism is not yet well established, being attributed to a defect in the dura mater that causes spinal cord herniation. 2

The first description of a case reporting this idiopathic condition was made by Wortzman in 1974. 3 The condition has higher incidence in females, usually in the intermediate decades of life, with a mean age of 49 years. 4 The most common clinical presentation is slowly progressive myelopathy, characterizing Brown-Séquard syndrome. 5 Surgical management is considered the best approach for symptomatic patients with ISCH, because it has favorable neurological outcomes and generally prevents further clinical deterioration. 6

The present article analyzed 62 cases of ISCH and revealed a balanced gender distribution, with a mean age of 49.94 years. Complete anterior herniation was the most prevalent (76.47%). The main symptoms included weakness in the lower limbs, back pain and Brown-Séquard syndrome. The most common treatment was laminectomy with duroplasty, showing significant improvement in the majority of patients.

The etiology of ISCH remains uncertain, with theories involving disturbances in neural development, inflammatory reactions of the dura mater, arachnoid cysts, and repetitive injuries. Surgery is widely recommended for symptomatic cases, with techniques such as widening the dural defect and dural grafting to prevent recurrences. Although there is no standardized procedure, most patients experience significant neurological improvement.

This report describes a case of idiopathic spinal cord hernia in a 45-year-old male patient with progressive myelopathy as a clinical manifestation. The diagnosis was confirmed by magnetic resonance imaging (MRI) and computed tomography (CT) myelography. A T2-to-T3 laminectomy, reduction of the ventral medullary hernia, and duroplasty were performed with successful completion of the procedure and complete resolution of the ventral medullary hernia.

## CASE REPORT

**Figure 1:**
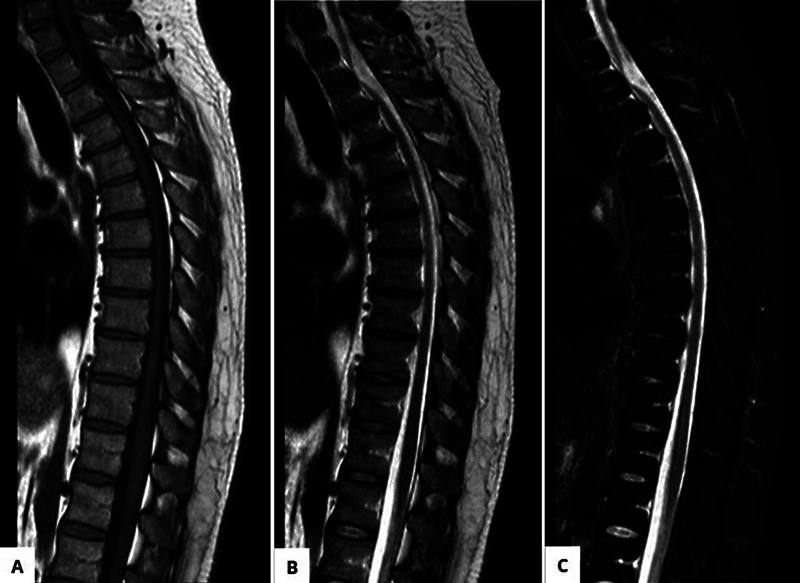
Magnetic resonance imaging (MRI) of the spine. ( **A** ) Sagittal T1. ( **B** ) Sagittal T2. ( **C** ) Sagittal STIR.

**Figure 2:**
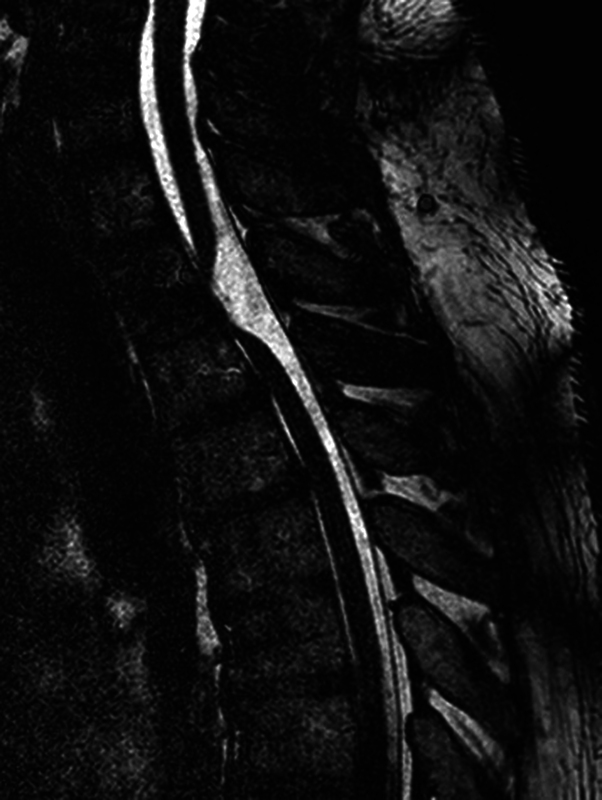
Magnetic resonance imaging (MRI) of the Spine – sagittal gradient echo.

**Figure 3:**
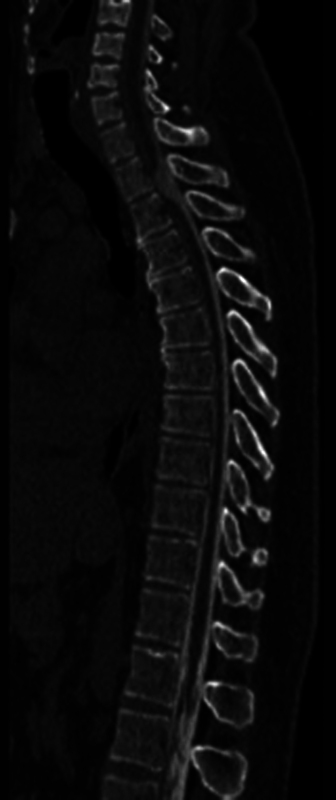
Computed tomography of the spine – sagittal bone window.

**Figure 4:**
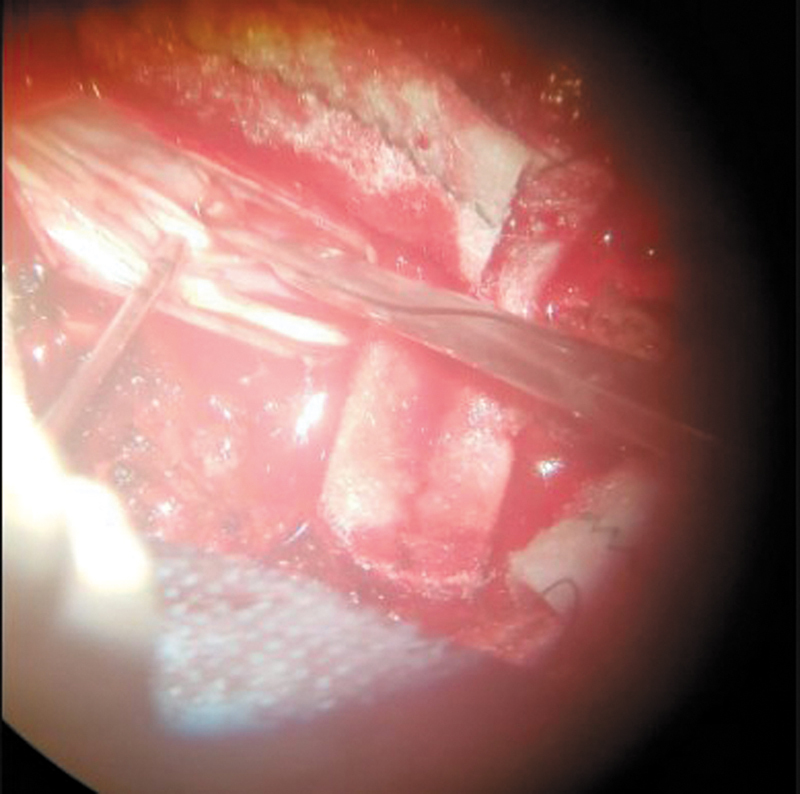
Intraoperative photograph.

A 45-year-old male patient presented to the emergency room complaining of progressive numbness for 4 months, with paraparesis that progressed to gait disorder. No history of trauma or previous spine surgery. The diagnosis was made by MRI and CT myelography ( Figures 1 , 2 , and 3 ), the findings were consistent with ventral medullary hernia at the T2-T3 level. The patient underwent a T2-T3 laminectomy, reduction of the ventral medullary hernia with cord release, and duroplasty to correct the dural defect ( Figure 4 ).

**Figure 5:**
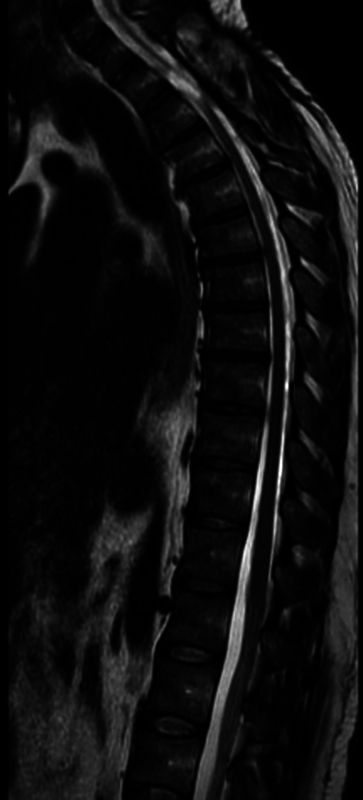
Post-surgical MRI of the spine– sagittal T2.

The patient showed improvement in symptoms and functionality for daily activities after surgical resolution. However, the patient did not fully recover his initial state. After 5 years, a postoperative follow-up MRI ( Figure 5 ) showed the absence of anterior torsion of the spinal cord, present in the preoperative images, indicative of complete resolution of the ventral medullary hernia.

## DISCUSSION

In **Table S1** ( **Supplementary Material – Table S1** –available at https://www.arquivosdeneuropsiquiatria.org/wp-content/uploads/2025/08/ANP-2025.0086-Supplementary-Material.docx ), the distribution between genders was nearly balanced among our 62 cases, with 30 males (48.39%) and 32 females (51.61%). The mean age of the patients was 49.94 years, ranging from 12 to 77 years, indicating that the condition can affect a wide age spectrum, from adolescents to elderly individuals.

The type of herniation was specified in 51 cases. Among these, complete anterior herniation was the most prevalent, accounting for 39 cases (76.47%), while anterolateral ones were observed in 12 cases (23.53%). In 11 cases (17.74%), the type of herniation was not specified.

These findings highlight the predominance of complete anterior herniation in this sample. The most frequent clinical manifestations included lower extremity weakness, back pain, Brown-Séquard syndrome, gait abnormalities, and sensory disturbances. These symptoms are consistent with typical findings of spinal cord or nerve root compression associated with disc herniation, underscoring the condition's significant functional impact. The most commonly performed surgical procedure was laminectomy combined with duroplasty using a graft or flap. The majority of patients who underwent this intervention experienced significant clinical improvement, supporting surgical treatment efficacy in alleviating symptoms and promoting functional recovery.

Spinal cord herniation can be classified as idiopathic, post-traumatic, and iatrogenic. 7 The etiology of the idiopathic one is postulated as arising from a congenital or acquired defect that predisposes to spinal cord protrusion. This origin was initially suggested by Wortzman et al. in 1974, 3 based on the conception of congenital dural defects triggering medullary herniation and the formation of a preexisting ventral meningocele. However, the pathogenesis remains largely undefined, with multiple theories documented in the literature to elucidate the mechanism underlying dural defect formation and subsequent herniation, including trauma, congenital dura mater deficiency, and pressure-related dural erosion secondary to intervertebral disc herniation. 8 There are four main theories that stand out: neural disturbance in medullary hernia, dural inflammatory reactions, arachnoid cyst in medullary hernia, and repetitive occult lesions.

### Theory of neural disturbance in medullary hernia

Bartels et al. 9 hypothesized through a neuropathological analysis that medullary hernia is not acquired, instead resulting from a developmental disorder in the first gestational trimester, in which neural crest cells cluster and differentiate into neural tissue rather than dura mater. Then, the clustering of nonfunctional neuronal cells adjacent to the spinal cord results in a defect in the dura mater. 9

### Theory of dural inflammatory reactions

Najjar et al. 10 propose that the pathogenesis consists of dural inflammatory reactions that cause adhesions between the medulla and dura mater, with development of erosion and formation of a dural defect. Then, progressive herniation of the spinal cord through the defect as a consequence of the mechanical stress of cerebrospinal fluid (CSF) pulsations. 10 This theory is reaffirmed by Yamamoto et al., who believe that adhesions around the dura mater caused by the inflammatory process may be the pathogenesis of idiopathic medullary hernia, evidenced and reported by the intraoperative finding of a dorsal subarachnoid septum. 11

### Theory of arachnoid cyst in medullary hernia

The medullary hernia would occur due to a congenitally present dorsal arachnoid cyst, which displaces the thoracic cord against the ventral dura mater, causing erosion and formation of a dural rupture. 12

### Repetitive occult lesions

The pathological basis is also attributed to repetitive occult lesions in the spine, which cause microdamage to the dura mater and progressively generate a dural defect. 13

The average age range of reported cases is usually around 50 years, with a more frequent incidence among women at an approximate ratio of 2:1. 14 15 16 The clinical picture is usually slow and progressive, sometimes stable, and in the initial period not all patients recognize symptomatic changes or, in some cases, these manifestations are initially attributed to other causes. This can delay diagnosis until more obvious symptoms, such as progressive difficulty in locomotion, frequent falls, and evolving paresthesia preceded by a feeling of warmth, are reported. 14

A meta-analysis performed in 2009, which included 126 cases, revealed that Brown-Séquard syndrome was the most common clinical manifestation, present in 66% of cases (n = 85), followed by paraplegia, observed in 30% of cases (n = 39), and isolated sensory (3%, n = 4) or motor (1%, n = 1) deficits. 16 This syndrome is characterized by paresis and loss of vibratory and positional sensation on the ipsilateral side, often accompanied by thermoalgesic hypoesthesia on the contralateral side.

A rare manifestation, but one that deserves to be highlighted, was documented by Mehta et al., 17 a case of ventral medullary hernia inducing diffuse subarachnoid hemorrhage and formation of intradural hematoma. 17 In the context of this case, the patient initially presented with nonspecific symptoms of chest pain, which complicated the diagnostic process.

Most of the reported idiopathic spinal cord hernias were found between T2 and T10, with a high predominance (79%) between T4 and T8. 18 The diagnosis is established through MRI, especially when high-resolution methods are used, such as steady-state axial constructive interference. 19 It is based on the typical finding of ventral displacement of the spinal cord in a “tent” shape, with subarachnoid space suffering obliteration in the anterior perimedullary area and enlargement in the dorsal one. 20

Differential diagnoses of idiopathic spinal cord herniation mainly include dorsal arachnoid cyst, astrocytoma, herniated disc, extradural compression, and transverse myelitis. 8 Arachnoid cysts have signal intensity similar to CSF on MRI images. A CT myelography is used to establish this differential diagnosis, allowing the distinction between the enlargement of the dorsal subarachnoid space and the dorsal arachnoid cyst, whose identification is made possible by filling with contrast media. 21

Surgical intervention is widely recommended for all symptomatic cases of spinal cord herniation. The main objective of this type of management is to release the medullary herniation, returning the medulla to the anatomical position and preventing reherniation. Studies indicate that the neurosurgical outcome for this condition is favorable in approximately 75.2% of cases, demonstrating significant clinical improvement. 22

Currently, there is no standardized procedure for spinal cord herniation intervention. Although there are reports of the anterior transthoracic approach, the most common is the posterolateral transpedicular approach, costotransversectomy, or decompressive laminectomy, the latter being the most recommended. 8 This approach allows for safer manipulation of the spinal cord. However, it may have limitations, such as the lack of clear visualization of the ventral dural surface without extensive retraction of the spinal cord, often requiring the release of dentate ligaments and adhesions to mobilize it. 23 This manipulation can result in postoperative weakness and unwanted neurological deficits.

Intraoperative ultrasonography has emerged as a useful tool to identify and confirm the level of location of spinal cord displacement prior to opening the dura mater. 24 25 There are two main techniques to repair the defect and prevent recurrence of the herniation.

First, enlargement of the dura mater defect, which is popular among Japanese authors, performed when there is duplication of the dura mater. 26 It consists of disengaging the spinal cord by cutting the inner layer of the dura along both sides of the herniated cord. Although technically easy and minimally invasive, in the literature it is more associated with the risk of CSF leakage in the anterior extradural region, leading some authors to prefer the anterior dural graft technique. 27 However, in their meta-analysis of 234 patients, Groen et al. found that the enlargement of the dural defect resulted in a significant clinical improvement (89.7%) when compared with the previous filling (70.5%), with a lack of reliable data on the prevalence or clinical relevance of CSF ventral extradural accumulation in the postoperative period. 22

Second, anterior dural grafting (duroplasty) consists of releasing the spinal cord and placing a filling in the space between the defect and the dura mater cord to prevent reherniation. It is preferred by most surgeons for requiring less extension in visualization of the dural defect and minimal traction of the marrow to insert the graft. Several materials have been suggested for filling, such as fascia, fat, expanded polytetrafluoroethylene, dural substitute, politetrafluorethylene, and bovine pericardium. 19

Due to the rarity of this pathology and the paucity of long-term data, no graft material has been shown to be superior to any other in preventing recurrent tying of the spinal cord. 28

The direct suture technique, although a viable option, demands some important considerations. While it may offer a straightforward approach to treating CSF leakage, it can result in increased compression on the spinal cord during the suturing procedure. 19 Additionally, it implies a strictly dorsal approach with a small window to access the ventral dura mater, which may predispose to extensive torsion of the medulla to create sufficient working space between it and the ventral defect. 26 29 In their literature review, Saito et al. found that around 20% of patients with direct dura mater closure worsened clinically after surgery, compared with the 10% who worsened with dural grafting and/or defect enlargement techniques. 29 30 31 32 33 34 35 36 37 38 39 40 41 42 43 44 45 46 47 48 49 50 51 52 53 54 55 56 57
